# CA19-9 as a marker for ovarian cancer: alone and in comparison with CA125.

**DOI:** 10.1038/bjc.1985.161

**Published:** 1985-07

**Authors:** P. A. Canney, P. M. Wilkinson, R. D. James, M. Moore


Br. J. Cancer (1985), 52, 131–133

### Short Communication

### CA19-9 as a marker for ovarian cancer: Alone and in comparison with CA125

P.A. Canney[1], P.M. Wilkinson[1], R.D. James[2] & M. Moore[3]

[1]*Departments of Clinical Pharmacology;* [2]*Radiotherapy and* [3]*Paterson Laboratories, Christie Hospital and Holt Radium Institute, Wilmslow Road, Withington, Manchester 20, UK.*

The monoclonal antibody 19-9 was originally raised against a human colon carcinoma cell line, SW1116 (Koprowski *et al.*, 1981). The antigen defined is a carbohydrate determinant of a circulating antigen designated CA19-9 (Del Villano *et al.*, 1983). A radioimmunoassay to detect CA19-9 in serum was recently developed (Del Villano *et al.*, 1983) and elevated levels of CA19-9 have been found association with a wide range of benign and malignant conditions (Del Villano & Zurawski, 1983) including ovarian carcinomas (Ricolleau *et al.*, 1983).

To date CA125 has shown the most clinical promise as a marker for ovarian tumours (Bast *et al.*, 1983; Canney *et al.*, 1984). The present study has investigated CA19-9 levels in the serum of patients with known ovarian carcinoma in whom CA125 levels were also recently measured. The object was to test CA19-9 as a serum marker alone and in comparison with CA125 to see if the pair of antigens provided more information than each one separately.

Sera of 55 patients with histologically proven ovarian adenocarcinoma, known to have persisted after laparotomy, or to have recurred after previous treatment, were examined for CA19-9 levels.

The sampling procedure, treatment methods and assessment of patients were as described in the first part of the study (Canney *et al.*, 1984).

CA19-9 was measured using a solid phase sandwich radioimmunoassay (International CIS, UK, Ltd., London). Sera were stored at $-20°C$ until assayed. The upper limit of normal was taken as $33\,u\,ml^{-1}$, a level exceeded by 1% of 260 presumed normal subjects (manufacturers information).

CA125 was also measured using a commercially available immunoradiometric assay (International CIS, UK Ltd) as previously described (Canney *et al.*, 1984).

Correspondence: P.A. Canney.
Received 1 February 1985; and in revised form 15 March 1985.

The sensitivity of the assay (defined as no. true +ve ÷ no. true +ve + no. false negative) for ovarian adenocarcinomas is shown in Table I. The overall sensitivity was 29% with a proportion of all the histological types being positive. A further two patients with granulosa cell tumours had CA19-9 levels $<33\,U\,ml^{-1}$. There was no correlation between elevation of serum CA19-9 and bulk of residual tumour, the antigen having been detected as frequently in patients with minimal residual disease (no lesion >2 cm) as in patients with gross bulk disease (any lesion >10 cm). (Table II).

Twelve patients whose initial CA19-9 level was elevated had serial levels performed during the course of their treatment. Changes in measured serum antigen level *versus* clinical response are shown in Figure 1. No patient who responded to treatment had a rising CA19-9 titre, and in all three cases the antigen level was within the normal range

**Table I** Frequency of detection of an elevated serum serum CA19-9 level in ovarian carcinoma overall and by histological type

| *Histology* | *Total* | *Positive* (>$33\,U\,ml^{-1}$) |
|---|---|---|
| Adenocarcinomas: | | |
| Serous | 24 | 4 |
| Mucinous | 8 | 4 |
| Endometroid | 7 | 4 |
| Undifferentiated | 11 | 2 |
| Clear Cell | 5 | 2 |
| | 55 | 16 |

**Table II** Sensitivity of CA19-9 relative to tumour bulk at presentation

| *Tumour Bulk* | *Total* | *Positive* (%) |
|---|---|---|
| <2 cm | 18 | 4 (18) |
| 2–10 cm | 13 | 5 (28) |
| >10 cm | 24 | 7 (23) |

© The Macmillan Press Ltd., 1985

**Figure 1** Changes in serum CA19-9 level by clinical course of the disease. Serum CA19-9 was measured before and at the completion of treatment.

at the conclusion of treatment. This is in marked contrast to those patients with static or progressive disease, none of whom showed a similar fall in CA19-9 serum level.

Overall 40 patients were available for assessment of response to chemotherapy of whom 23 (58%) responded. The number of responses by result of the marker assays is shown in Table III. The proportion of responders (23%) in the $CA19\text{-}9^+$ group was significantly lower than in the $CA19\text{-}9^-$ group (74%) ($\chi^2 = 9.34$; $P < 0.01$).

**Table III** Patient response to chemotherapy by presence or absence of CA125 and CA19-9

| | $CA19\text{-}9^+$ | $CA19\text{-}9^-$ | $CA125^+$ | $CA125^-$ |
|---|---|---|---|---|
| Responders | 3 | 20 | 16 | 7 |
| Failures[a] | 10 | 7 | 15 | 2 |

[a]Failures include patients with both static and progressive disease.

Comparison of the sensitivities of CA125 and CA19-9 is shown in Table IV. The overall sensitivity for CA125 was 76% in this group of patients, whilst the combined sensitivity, either CA125 or CA19-9 positive, rose to 80%.

For monitoring the course of the disease, variations in CA19-9 levels corresponded to those in CA125 levels in 8 cases, and reflected the clinical situation accurately. In three cases the serial changes in CA19-9 levels provided a better correlation with the eventual clinical course of the disease than changes in CA125 levels. In all three instances an initial partial response to chemotherapy was quickly followed by disease progression, before treatment was completed. CA125 levels fell initially, to within the normal range in two cases, before rising as disease progressed. However, CA19-9 levels in all three cases remained static or rose from the start providing a better indicator of eventual response to chemotherapy (Figure 2).

In one case an initial normal CA19-9 level rose during disease progression, whilst CA125 remained negative throughout.

**Table IV** Combined sensitivity of CA19-9 and CA125

| *Assay Result* | *No. of patients* (%) |
|---|---|
| $CA19\text{-}9^-/CA125^+$ | 28 (51) |
| $CA19\text{-}9^+/CA125^-$ | 2 (4) |
| $CA19\text{-}9^+/CA125^+$ | 14 (25) |
| $CA19\text{-}9^-/CA125^-$ | 11 (20) |

**Figure 2** Comparison of serial CA19-9 (○) and CA125 (●) levels in three patients during the course of chemotherapy. (---) $35\,U\,ml^{-1}$.

When examined immunohistochemically CA19-9 has been shown to occur in association with ovarian adenocarcinomas (Charpin *et al.*, 1982) the mucinous histological types reacting much more frequently than serous types. CA19-9 has been shown to be secreted into the serum in ovarian cancer patients (Ricolleau *et al.*, 1983) but results of serum assays have not previously been reported in detail. The sensitivity of the CA19-9 assay, 29%, is not adequate for it to be used alone as a marker for ovarian tumours. However, a raised CA19-9 level before chemotherapy does appear to be a significant adverse prognostic factor. This was not related to the presence of bulk disease, as the percentage of patients with elevated CA19-9 levels was independent of the bulk of disease. No such prognostic significance was apparent for the CA125 antigen in the series.

When the CA19-9 level was elevated there was a good correlation with the clinical course of the disease, in those patients in whom serial levels were measured. In three instances this was more evident than the serial changes in CA125 level, which fell initially before rising again as the disease progressed.

CA125 is the ovarian antigen which has shown the most clinical promise to date (Bast *et al.*, 1983; Canney *et al.*, 1984). However, it seems likely that no tumour associated antigen *per se* will give useful information in all patients, not least because expression is frequently heterogeneous in a given neoplasm, regardless of the histological appearance of the cells (Kabawat *et al.*, 1983). However, the concept of using more than one marker is well established in the deployment of HCG and AFP to monitor testicular tumours. The use of a second marker, could theoretically increase serological detectability, if the substance were secreted by additional subpopulations of neoplastic cells. The efficacy of monitoring response to therapy would thereby be increased. The "worst case" serial marker results most accurately reflect the eventual response in the case of HCG and AFP, and a similar situation was evident in 4 patients in our series. Differential secretory activity of cells expressing CA125 and CA19-9 could explain, at least in part, the varied changes in serial levels observed in response to chemotherapy and this interpretation could be amenable to evaluation by immunohistochemistry. The present data are consistent with the view that CA19-9 secretion is predominantly a property of the more malignant, or drug resistant, cells within the carcinomas studied in this series.

The specificity (false positive rate) will be worsened by using a pair of markers but this is not relevant once a histological diagnosis has been made.

Apart from its prognostic significance, measurement of CA19-9 gave additional information to CA125 in 6 cases (11%), including the two patients who were CA19-9$^{+}$ but CA125$^{-}$, although the sensitivity of CA125, at 76%, was slightly lower in this particular group of 55 patients than in previously reported series (Bast *et al.*, 1983; Canney *et al.*, 1984) and in all patients who have had CA125 levels measured at this institute where the overall sensitivity is currently 81/97 (83%). However, at present no other antigenic marker is as readily available as an alternative or addition to CA125 and an initial CA19-9 assay could be of some clinical value.

### References

BAST, R.C. Jr., Klug, T.L., ST JOHN, E. & 9 others. (1983). A radioimmunoassay using a monoclonal antibody to monitor the course of epithelial ovarian cancer. *N. Engl. J. Med.*, **308**, 883.

CANNEY, P.A., MOORE, M., WILKINSON, P.M. & JAMES, R.D. (1984). Ovarian cancer antigen CA125: A prospective clinical assessment of its role as a tumour marker. *Br. J. Cancer*, **50**, 765.

CHARPIN, C., BHAN, A.K., ZURAWSKI, V.R. Jr. & SCULLY, R.E. (1982). Carcinoembryonic antigen (CEA) and carbohydrate determinant 19-9 (CA19-9) localization in 121 primary and metastatic ovarian tumours; An immunohistochemical study with the use of monoclonal antibodies. *Int. J. Gynecol. Pathol.*, **1**, 231.

DEL VILLANO, B.C. & ZURAWSKI, V.R. Jr. (1983). The carbohydrate antigenic determinant 19-9 (CA19-9): A monoclonal antibody defined tumour marker. In *Immunodiagnostics*, (Eds. Aloisi & Jayson Hyun), Alan R. Liss Inc.: New York.

DEL VILLANO, B.C., BRENNAN, S., BROCK, P. & 8 others. (1983). Radioimmunometric assay for a monoclonal antibody-defined tumor marker, CA19-9. *Clin. Chem.*, **29**, 549.

KABAWAT, S.E., BAST, R.C. Jr., WELCH, W.R., KNAPP, R.C. & COLVIN, R.B. (1983). Immunopathologic characterization of a monoclonal antibody that recognises common surface antigens of human ovarian tumours of serous, endometroid and clear cell types. *Am. J. Clin. Pathol.*, **79**, 98.

KOPROWSKI, H., HERLYN, M., STEPLEWSKI, H.H. & SEARS, H.F. (1981). Specific antigen in serum of patients with colon carcinoma. *Science*, **212**, 53.

RICOLLEAU, G., FUMOLEAU, P., KREMER, M., CURTET, C., DOUILLARD, J.Y. & CHATAL, J.F. (1983). Radioimmunoassay of the CA125 antigen in epithelial ovarian carcinomas: Advantages as compared with CA19-9 and CEA. *Proc. XI Annual Meeting, International Soc for Oncodevelopmental Biology and Medicine. Stockholm.*


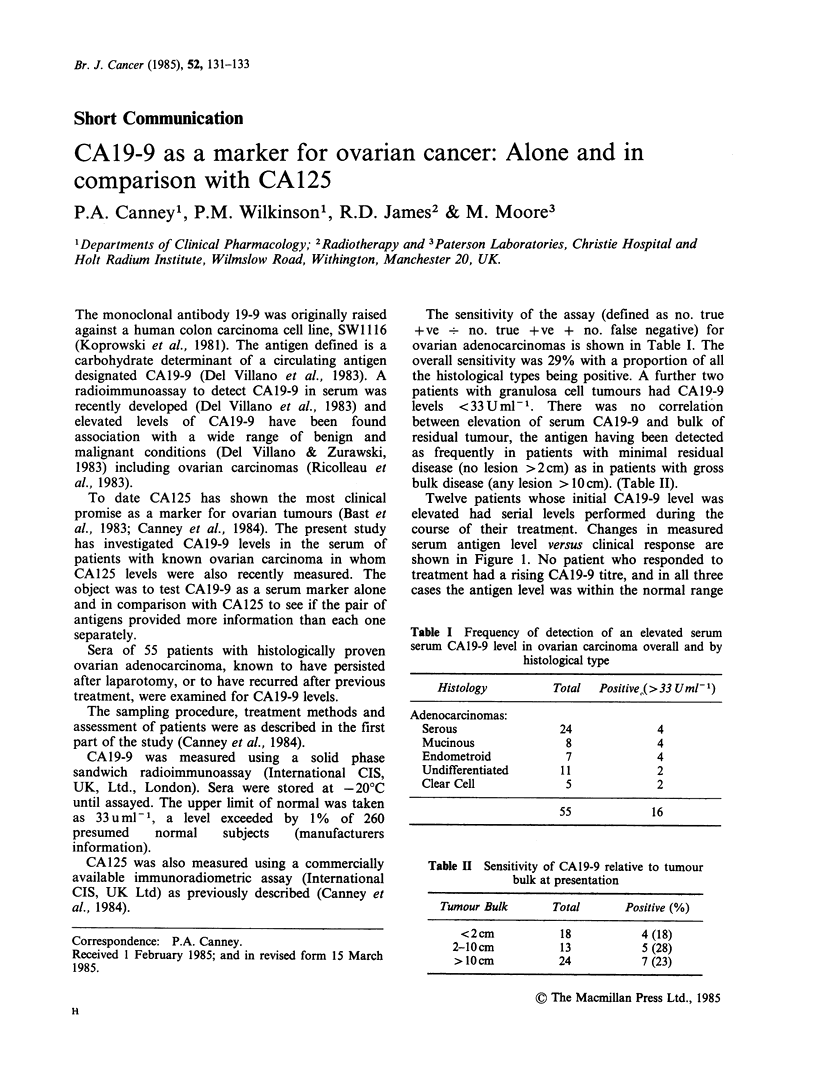

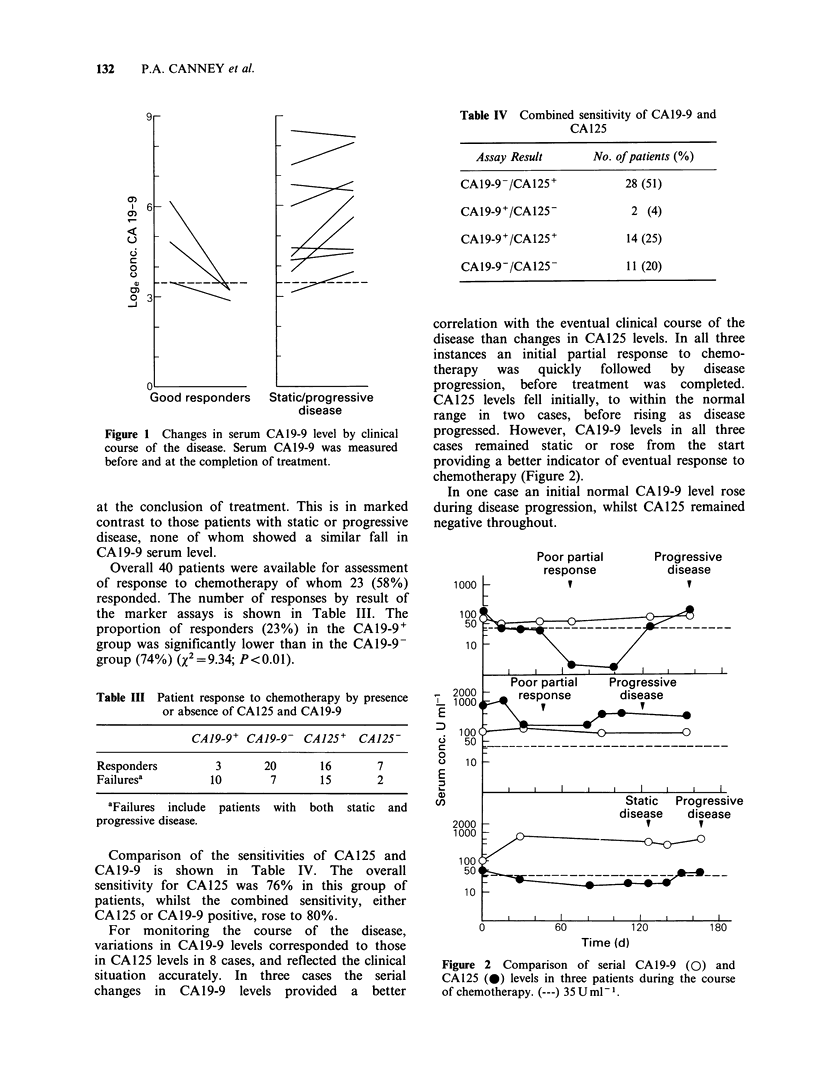

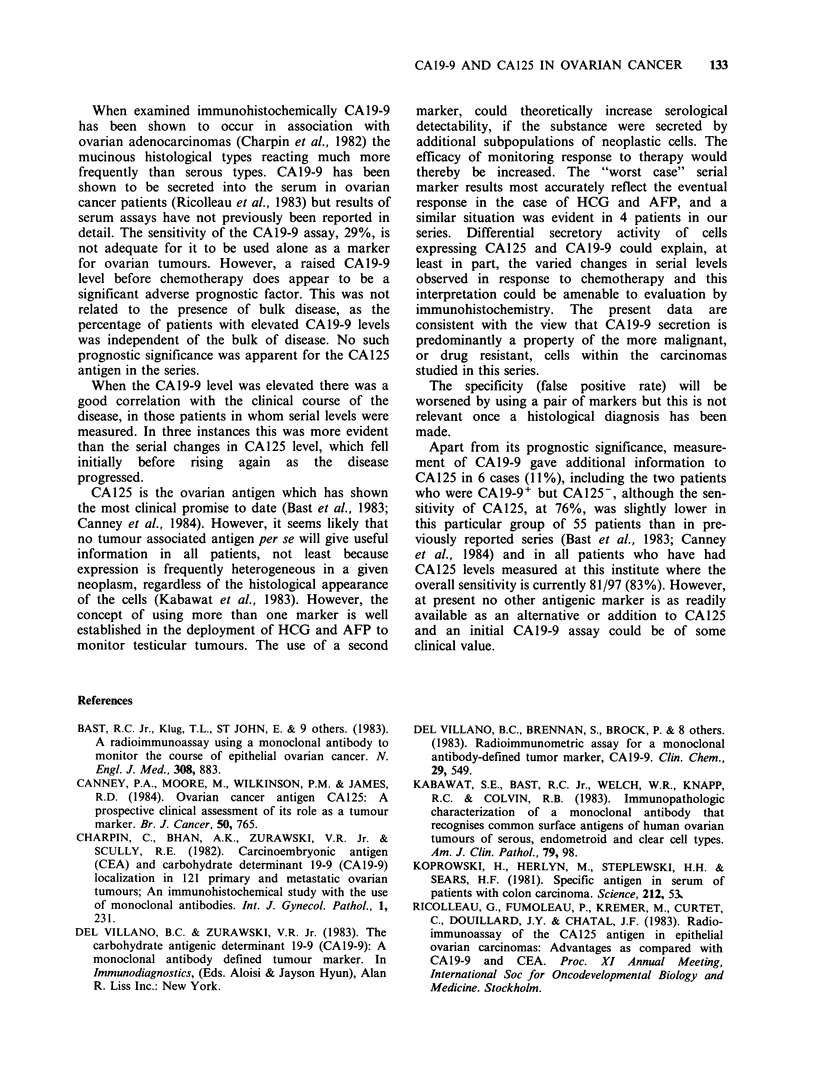


## References

[OCR_00354] Bast R. C., Klug T. L., St John E., Jenison E., Niloff J. M., Lazarus H., Berkowitz R. S., Leavitt T., Griffiths C. T., Parker L. (1983). A radioimmunoassay using a monoclonal antibody to monitor the course of epithelial ovarian cancer.. N Engl J Med.

[OCR_00360] Canney P. A., Moore M., Wilkinson P. M., James R. D. (1984). Ovarian cancer antigen CA125: a prospective clinical assessment of its role as a tumour marker.. Br J Cancer.

[OCR_00366] Charpin C., Bhan A. K., Zurawski V. R., Scully R. E. (1982). Carcinoembryonic antigen (CEA) and carbohydrate determinant 19-9 (CA 19-9) localization in 121 primary and metastatic ovarian tumors: an immunohistochemical study with the use of monoclonal antibodies.. Int J Gynecol Pathol.

[OCR_00382] Del Villano B. C., Brennan S., Brock P., Bucher C., Liu V., McClure M., Rake B., Space S., Westrick B., Schoemaker H. (1983). Radioimmunometric assay for a monoclonal antibody-defined tumor marker, CA 19-9.. Clin Chem.

[OCR_00388] Kabawat S. E., Bast R. C., Welch W. R., Knapp R. C., Colvin R. B. (1983). Immunopathologic characterization of a monoclonal antibody that recognizes common surface antigens of human ovarian tumors of serous, endometrioid, and clear cell types.. Am J Clin Pathol.

